# Best Practices for Microbiome Study Design in Companion Animal Research

**DOI:** 10.3389/fvets.2021.644836

**Published:** 2021-04-09

**Authors:** Jessica K. Jarett, Dawn D. Kingsbury, Katherine E. Dahlhausen, Holly H. Ganz

**Affiliations:** AnimalBiome, Oakland, CA, United States

**Keywords:** experimental design, clinical trials, feeding trials, diet trials, recruitment, microbiome testing

## Abstract

The gut microbiome is a community of microorganisms that inhabits an animal host's gastrointestinal tract, with important effects on animal health that are shaped by multiple environmental, dietary, and host-associated factors. Clinical and dietary trials in companion animals are increasingly including assessment of the microbiome, but interpretation of these results is often hampered by suboptimal choices in study design. Here, we review best practices for conducting feeding trials or clinical trials that intend to study the effects of an intervention on the microbiota. Choices for experimental design, including a review of basic designs, controls, and comparison groups, are discussed in the context of special considerations necessary for microbiome studies. Diet is one of the strongest influences on the composition of gut microbiota, so applications specific to nutritional interventions are discussed in detail. Lastly, we provide specific advice for successful recruitment of colony animals and household pets into an intervention study. This review is intended to serve as a resource to academic and industry researchers, clinicians, and veterinarians alike, for studies that test many different types of interventions.

## Background and Significance

The gut microbiome is a complex and diverse community of bacteria, viruses, fungi, and other microorganisms that colonize the digestive tract in animals, including both humans and pets. Whipps et al. (1) were the first to define the term microbiome: “a characteristic microbial community occupying a reasonably well-defined habitat which has distinct physio-chemical properties. The term thus not only refers to the microorganisms involved but also encompasses their theaters of activity.” Because some confusion subsequently arose in the rapidly expanding microbiome field in regards to the origins and definition of microbiome and how it differs from microbiota, the following clarifications were recently proposed to amend the original definition (2): “The microbiome not only refers to the microorganisms involved but also encompass their theater of activity, which results in the formation of specific ecological niches. The microbiome, which forms a dynamic and interactive micro-ecosystem prone to change in time and scale, is integrated in macro-ecosystems including eukaryotic hosts, and here crucial for their functioning and health. The microbiota consists of the assembly of microorganisms belonging to different kingdoms [Prokaryotes (Bacteria, Archaea), Eukaryotes (e.g., Protozoa, Fungi, and Algae)], while “their theater of activity” includes microbial structures, metabolites, mobile genetic elements (e.g., transposons, phages, and viruses), and relic DNA embedded in the environmental conditions of the habitat.” Gut microbiota can play key roles in many areas of host health, including development, digestion, behavior, immune system function (3–6). Alterations in the composition of the gut microbiome have also been associated with certain health conditions, including obesity (7, 8), diabetes (9, 10), kidney disease (11, 12), skin disorders (13), chronic enteropathy (14–16), immune mediated disorders (17, 18), and allergies (19).

There are many factors that influence the composition of the gut microbiome, and given how important it is for animal health, it is pertinent to understand the effects different interventions have on the microbiota. For example, diet has a strong impact on the gut microbial community, and therefore can influence numerous aspects of animal health. The diet is the substrate for microbial growth, which enables the production of microbial metabolites including short chain fatty acids (SCFAs), amino acids, vitamins, and secondary bile acids (20, 21). Many of these metabolites, particularly SCFAs, are associated with an array of physiological effects on the host (5, 22). Current research is beginning to explain how the abundance of specific taxa and metabolites can be manipulated by altering the diet (5, 23–26), and in conjunction how some taxa and metabolites are linked to several health conditions. However, a more detailed understanding of how diet and nutrition affect the composition of microbiota, and how nutrition-based influences on the microbiota affect overall health are necessary first. Medications, supplements, and other interventions can also impact the microbiota, which is why we stress the importance of microbiome testing for clinical trials and food trials.

Understanding the role of an intervention on gut microbial health can have a far-reaching impact from veterinary medicine, the pet food and supplement industry, to consumer choices. For example, the aforementioned microbiome-associated health problems can be expensive and difficult to treat in companion animals, as well as greatly compromise the performance of animals engaged in work or competition (6, 27–30). Specialized interventions that target microbial taxa associated with specific disorders are a plausible method to prevent and more effectively treat the root cause of some of these conditions but require further study and development.

Research into what affects companion animal microbiota is carried out by a variety of commercial and academic entities, but access to findings is limited for several reasons. While some findings are published in the scientific literature, many others remain proprietary, or are unable to meet the standards for publication in a peer-reviewed journal. It is in the best interest of the companion animal scientific and commercial community as a whole to promote publication and open sharing of the results of robustly designed and analyzed trials and microbiome studies. Some parties who are willing and able to fund and conduct such studies may lack the necessary expertise to design effective trials that will yield the high-quality data sought.

Recommendations for best practices for the design and execution of feeding trials and clinical trials in companion animals where the gut microbiota will be assessed are given. Classical approaches that are appropriate for traditional nutrition or clinical studies (for example, digestibility studies) are not necessarily suitable for assessing the microbiota. Further, there is a need to address common mistakes that have led to underpowered studies that neither meet standards for publication nor answer the desired questions, which could have been prevented with small changes to the experimental design or recruitment choices. This review is intended to apply to trials for diets, medications, non-surgical treatments, and dietary supplements; the term “intervention” will be used to refer to these treatments collectively. While an emphasis is placed on diet and nutrition throughout, recommendations can be generalized for all types of interventions, and comments on special considerations for clinical studies are made where appropriate.

Here we summarize the best practices for conducting gut microbiome studies as part of clinical and feeding trials in companion animals. We cover experimental design, dietary considerations, recruitment, and microbiota sampling choices; we provide suggestions for more specialized or comprehensive reviews on specific topics where these exist. This review touches on a handful of basic and commonly used designs in intervention trials and microbiome studies, with general recommendations and caveats for each. Beyond providing a brief overview of the two most common sequencing methods currently used to characterize microbial communities, we do not provide a comprehensive review of the diverse methods for sequencing or analyzing microbiome data, but rather refer readers to the many excellent reviews on this topic, including several written for non-bioinformaticians (31–33).

## Experimental Design

The importance of a robust study design cannot be overstated. Small budgets, short time frames, and a limited number of subjects are just a few of the restrictions that researchers can face, but with a good study design, these obstacles can be navigated. Numerous projects have lost time, money, and effort due to inconclusive data that a strong experimental design could have prevented. This section outlines several basic and commonly used types of interventional designs for microbiome studies, with general recommendations and caveats for each. For more comprehensive information on experimental designs, including purely observational study designs which are not discussed here, the reader is referred to the extensive literature on the subject (34–36), particularly for microbiome and nutrition studies (37, 38).

Methods used to characterize the composition and function of gut microbiota continue to advance. While traditional cultivation methods fail to capture the full diversity of microbes (39), they continue to have relevance for screening for pathogens as well as antimicrobial resistance. Advancements in culture-independent approaches have led to several types of high-throughput sequencing methods. Which sequencing method to use for a study depends on many factors. Compared here are two of the most common sequencing methods used for microbial community characterization: amplicon sequencing and shotgun metagenomic sequencing.

Amplicon sequencing is a method of sequencing specific genes that are amplified using the Polymerase Chain Reaction (PCR), followed by DNA sequencing of the PCR product or amplicon. When amplicon sequencing is used for microbial characterization, PCR primers are designed for one or more of the hypervariable regions of the bacterial 16S ribosomal RNA (rRNA) gene (V1-V9) for bacteria and archaea, 18S rRNA gene for eukaryotes, or nuclear ribosomal internal transcribed spacer (ITS) for fungi. Shotgun metagenomic sequencing yields sequence data for all genomic DNA in a sample, including DNA viruses, fungi, protists, bacteria, archaea, as well as host DNA. Amplicon sequencing provides genus-level (for sequences of a subset of regions of the 16S rRNA gene using a short read sequencing technology such as Illumina) or species-level (for sequences of the full length 16S rRNA gene, which can be accomplished with long read sequencing technologies such as Pacific Biosystems and Oxford Nanopore). Depending on sequencing depth, metagenomic sequencing provides species or strain-level taxonomic resolution. Amplicon sequencing is significantly less expensive than the cost of metagenomic sequencing. And given the smaller dataset generated, amplicon sequences are also less costly to store and analyze from a computational perspective. Current databases for 16S rRNA sequences are robust for determining taxonomic identifications with amplicon and metagenomic sequencing yielding consistent bacterial community characterizations (40). Databases for shotgun metagenomics allow for far more detailed analyses, including genes associated with metabolic functions and antimicrobial resistance. The appropriate sequencing method to use depends on the study goals. There are other factors and sequencing methods to consider in addition to the ones mentioned here (41).

### Study Goals

It is paramount to carefully consider and tightly focus the aim or aims of the study. Trying to answer too many questions in a single study is a common mistake, especially when budgets are small. If the objectives are too broad ranging, the design may become overly complex and/or underpowered for sub-cohort groups. A design can effectively address multiple questions; however, studies with multiple goals will require more animals, more samples, a longer time course, or sometimes all three, which increases costs. Cost cutting on any of these aspects can potentially result in an underpowered design. In our experience, the most common experimental “victims” of this downsizing are control groups, control diets, and crossover aspects of the design.

Here is an example to illustrate this point: A researcher wants to study the effect of a novel protein source on the microbiota in not only healthy dogs, but also dogs with atopic dermatitis, a skin disorder associated with microbial imbalances (42, 43). The researcher plans to feed the novel protein source to N (number of) healthy dogs and N dogs with atopic dermatitis, and measure the microbiota changes in each group. Because the budget is small for the study, the researcher uses a very small number of dogs in each category, which causes the results to be statistically inconclusive. A better approach might be to limit the study of the novel protein to a larger group (N × 2) of only healthy dogs, and if the results are conclusive, to later conduct a separate study to see if the findings can be generalized to dogs with atopic dermatitis. In most situations, it is usually best to address a single question and use a simpler design.

#### Controls

Controls are crucial for good experimental design and generating interpretable results, but are unfortunately sometimes omitted in cost reduction scenarios. Controls allow the attribution of changes observed in the treatment group(s) to the applied intervention, when confounding variables are also accounted for (34, 35). It is common to use control interventions and control groups of animals when designing a study. Consistent differences in the intervention between individuals in both the control and treatment groups are more likely to yield an interpretable response. For example, if the effects of a high-fiber diet are being tested in pets, the control group should receive a single diet with a consistent level of fiber, rather than having pets remain on a varied set of previous diets with different levels of fiber.

There are several considerations when deciding which controls to use when testing an intervention's effect on a health condition. Especially for clinical trials, controls are necessary to account for health conditions that are intermittent or may spontaneously resolve. The type of control chosen depends on the health status of the animals in the study and the expected effect of the intervention. For healthy animals, an inactive placebo (see “Blinding” below) or no intervention may be a suitable control. However, in the case of unhealthy animals, this is typically not ethically acceptable if there is an effective alternative course of treatment (34, 36). In these cases, the experimental intervention can be compared to a standard course of treatment or an active control, rather than to no treatment. For example, in a recent study of dogs with chronic enteropathy, the microbiomes were compared in dogs that responded well to a hydrolyzed diet, those that required antibiotics for remission, and those that required both antibiotics and steroid treatment (16). By allocating dogs to each of these groups in a progressive fashion based on their individual response, every dog in the study received an effective treatment, at the same time forming the control group of dogs that was responsive to diet alone.

One key difference between microbiome studies and classical clinical or nutrition trials is that microbiome studies demand controls at not just the treatment level, but also at the technical level. Negative controls specific to microbiome studies such as sampling, extraction, and PCR blanks, and positive controls like mock communities help to control technical variability and standardize analyses (37, 44, 45). We refer readers to these sources (which further reiterate the importance of controls) for designing microbiome studies (32), analyzing microbiome data (33), and interpreting microbiome data (31).

### Basic Designs

#### Parallel Designs

**Figure 1:**
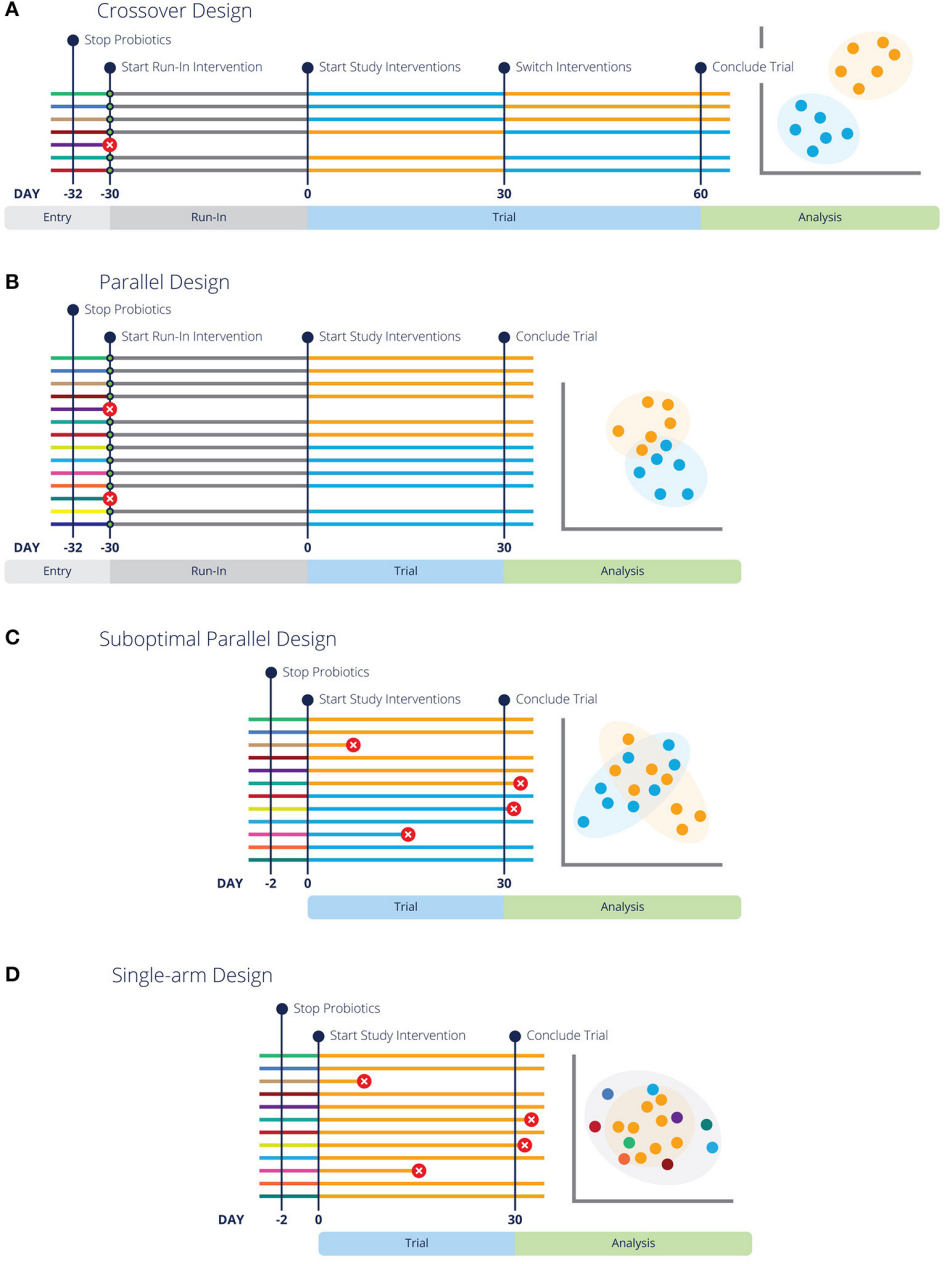
Different experimental design strategies for feeding trials or clinical trials are more likely to have conclusive results with run-in periods and pre-screening. Each line represents a different subject in the designated trial, with colors representing respective baseline, run-in and study interventions. Green circles indicate pre-screening tests. Lines with a red circle indicate the subject is rejected by screening criteria or did not complete the trial. The analysis graphic resembles a PCoA plot (a common representation of microbiome data), with each data point representing an individual in the study. Data points are colored by the diet or intervention at the time of sampling. **(A,B)** Illustrate optimal crossover and parallel study design, highlighting that entry screening and run-in periods lead to fewer dropouts and more conclusive results. Additionally, **(B)** Highlights the need for more subjects in a parallel design study. **(C)** Represents the same parallel design as **(A)**, but without pre-screening or a washout period, which lends to inconclusive results. Similarly, **(D)** illustrates a “Before vs. After” design without pre-screening or a washout period and inconclusive results.

Parallel designs are a simple and traditional type of experimental design where animals are assigned to specific groups or treatments, and remain in these groups for the duration of the experiment (Figures 1B,C). These are sometimes also called “between subjects” designs, because the subjects in the treatment group(s) are compared to different subjects in the control group (46). Parallel designs should always include a control group, unless precluded by ethical considerations (see “Controls” above) (34).

Unfortunately, uncontrolled single-arm studies or so-called before-after designs or “quasi-experimental” designs (47, 48) are also commonly used (Figure 1D), usually in an attempt to reduce study costs. In regard to microbiome studies, a typical uncontrolled design is where the microbiota are sampled in a single group of animals before a treatment (e.g., a diet change) and again some period of time afterwards, with no control group. While gut microbial communities do have higher within-individual correlation than between-individual correlation (49, 50), which would tend to favor comparisons within an individual, they are not static. Therefore, uncontrolled designs should be avoided whenever possible because bias is a serious problem in such designs (47).

Likewise, if any health problem or symptoms are being assessed in the experiment, these may also change or evolve naturally over time (51), and such changes can be easily mistaken for treatment effects in the absence of a control. For example, dogs with dermatitis may show symptomatic improvements due to seasonal changes in levels of environmental allergens, rather than an experimental diet (52). Together, these issues mean that even when significant results are obtained in an uncontrolled study, there is a substantial risk that later controlled studies may find a different result (35). If for ethical reasons a control group cannot be used in a parallel design (53), we recommend at minimum to increase the number of animals in the study.

#### Crossover Designs

Crossover designs are longitudinal studies in which each animal experiences all treatments or diets and thus serves as its own control (54–56) (Figure 1A). In this type of design, the same animal is sampled multiple times, and it can be a powerful approach for several reasons. First, the possibility of confounding due to differences between the experimental groups is eliminated (46, 56). Second, microbiomes tend to be more consistent within an individual than between individuals, so a crossover approach will typically have lower variance (49, 50).

Care should be taken in the design phase to try to prevent carryover effects or lingering effects from the previous intervention because these are challenging to correct for in analysis (55). First, all variables of the intervention and any health conditions being studied need to be considered. In addition to ethical concerns discussed above for conditions that are treatable or curable, interventions that may cure or permanently alter the condition present experimental problems. Treatments are given in a different order to each experimental group (Figure 1A), so if one group of animals receives a curative treatment in the first phase of the experiment, there will be no effect of the control in a subsequent phase. For example, clearing an animal of parasitic infection, performing a surgical procedure, or altering immune functions with treatments such as vaccines, will be guaranteed to have carryover effects. Crossover designs are best suited to studying chronic problems, and are particularly ideal for diet research (55, 56).

If there is reason to suspect that carryover will be an issue, switching to a parallel design, adding a run-in period, or extending the treatment period as appropriate are all preferable to trying to correct carryover effects in the analysis phase (55–57). A washout is a period without treatment or with a control treatment that allows the effects of a previous intervention to wane, and its length should be based on what is known about the duration of effect of the intervention (55). A washout period isn't necessary for trials that study the effects of interventions intended for a permanent result, such as curative or immunomodulatory treatments. Longer treatment periods may be necessary to reduce carryover effects to acceptable levels, such as if the diet change is substantial or pre-trial diets are varied, for example. This may not be necessary for interventions that are expected to produce a smaller effect (see “Timing of Sampling” for further detail). In dogs, carryover effects from the previous diet were not detectable either 4 weeks or 6 weeks after a diet change (58), so we suggest 4 weeks as an adequate diet trial duration, or washout period after a diet change.

### Sample Size

A frequent problem that we see in microbiome diet trials is small sample sizes (too few animals), leading to low power and inconclusive results. When calculating the appropriate sample size for a study, animals should be used as the experimental units, not the samples per animal. Unfortunately, calculation of effect size and statistical power remains technically challenging in microbiome studies (44, 59, 60), although several approaches have been proposed for assessing differentially abundant microbes (61, 62) and overall community composition (63). Reviewing the literature for similar study designs can be helpful in estimating the expected effect size and thus the approximate number of animals needed for a particular study. It is ideal if these studies were conducted in the same host species, but other host species can also provide useful information if the intervention was similar. For example, a study that found only small effects of cricket protein on the gut microbiota of humans (64) was used to inform the design of a study on cricket-based dog food (65). Crossover designs may be a good choice if the number of available animals is small or recruitment is difficult, because about twice as many individuals are recommended for an equivalent parallel design (Figure 1A vs. Figure 1B) (46, 56, 57).

### Blinding

Blinding is commonly used in controlled clinical trials of medicines or other treatments, and is sometimes relevant and recommended in feeding trials also. The chief benefit of this practice is in studies where a subjective metric is used, for example a visual assessment of skin condition, a quantification of the energy level of an animal, or generally whenever the presence, absence, or severity of clinical signs is reported. In these cases, the human reporters are subject to unconscious bias if they are aware of the diet or treatment, so blinding them to the intervention is desirable. In studies where only objective measurements are taken, such as body weight and microbiome composition, blinding is not strictly necessary but is nonetheless a good practice. Blinding for interventions typically involves a placebo, which should resemble the treatment in appearance and administration as much as possible. For feeding trials, thoughtful design of the study diets and their packaging can be used to blind the study participants. For example, if a new blend of dietary fiber is being tested in dogs with fiber-responsive diarrhea, it is preferable to manufacture two complete diets, one with the fiber and one without that are both presented in blinded packaging, rather than having participants mix in a fiber additive to their current food and thus being made aware of which treatment the animal is receiving. If an obvious dietary intervention cannot be avoided for practical reasons, and subjective response variables are being assessed, use of a blinded placebo is considered best practice.

#### Randomization

Randomization is a critical step in experimental design, and a common area for well-intentioned mistakes that can compromise a study. Randomly assigning animals to experimental groups is necessary to prevent bias that can arise when experimenters or pet owners are permitted to choose the treatment for each animal (66). Additionally, animals can vary in factors such as age, breed, body condition score, etc. that have documented associations with gut microbiota health (67–70). Assignment to groups should take place after animals are enrolled in the study, rather than as a condition of enrollment, so-called blinded randomization (36). Several general strategies for effective randomization exist, including simple random assignment, block randomization to ensure that the number of animals in each group is approximately equal when study enrollment happens over an extended period of time, and stratified randomization to ensure that groups are balanced with respect to characteristics such as sex and breed (71). We refer readers to Altman and Bland (71) and references therein for a more detailed explanation of these strategies and their implementation.

### Timing of Sampling

The timing of sample collection relative to interventions and dietary changes is important in microbiome studies, and best practices may differ from recommendations for clinical trials or classical animal nutrition studies. Doubling times for many species of microbes living in the mammalian gut are short (on the order of minutes to hours) and the transit of fecal material through the gut purges much of the community biomass on a daily or near-daily basis (72), so changes in the microbiota can occur on very short timescales (73). Despite this, communities within an individual tend to be stable over time and resist large-scale changes with short-term interventions (74–77). For example, microbiota do not seem to be permanently altered by even long-term dietary changes; once the dietary intervention is removed, the observed microbiota changes do not persist (58, 73). To ensure that community shifts observed in trials are not merely due to short-term volatility or variability, we recommend sampling at least 4 weeks after an intervention change, while the intervention of interest is still being administered to the animal (58). The mechanism and duration of action of the intervention should be taken into account alongside the general 4-week guideline.

For some interventions or life stages, sampling on a shorter and more frequent timescale may be appropriate. If the speed of the desired effect is an important product attribute, for example a diet intended to ameliorate diarrhea, observing a response that is both rapid and persistent may be important [for example, (14)]. Studies with interventions intended for the development of young animals, pregnancy, or lactation should conduct sampling at more frequent intervals than diets for adult animals, because there are substantial shifts in the microbiota that occur over short time periods during these developmental phases (4, 78, 79).

### Multiple Interventions

Medications, supplements, and probiotics, whether administered as part of the study protocol or not, may present unanticipated pitfalls when collecting samples for microbiome testing. While these interventions should be considered in recruitment as well (see “Recruitment”), it is important to account for and understand how other interventions may affect the study outcome. For example, non-study-related antibiotics should be avoided during microbiome studies and for at least several weeks prior to the beginning of a study because microbiota do not completely recover within this timeframe (80, 81). Several other common medications including antiparasitics (82) and proton pump inhibitors (83, 84) can also affect the microbiota and their use should be avoided where possible, or at least recorded. With the exception of antibiotics, most supplements and medications have not been tested for their interactions with the microbiota and should be avoided where possible. At a minimum, all medication, supplement, and probiotic usage and timing should be recorded and disclosed.

Probiotic supplements can affect which microbiome testing method can be used and should be considered when sampling microbiomes. A very common method of microbiome testing is based on PCR amplification and sequencing of the 16S rRNA gene and targets bacteria and archaea (37). DNA from bacterial probiotics can be detected in fecal samples (85), where it can overwhelm the signal from the native gut microbiota in 16S rRNA sequencing approaches. Fungi and other eukaryotes can also be detected with PCR amplification of the internal transcribed spacer (ITS) of the 18S rRNA. Eukaryotic probiotics, such as yeasts, can also be detected in fecal samples and overwhelm the sequencing signal of ITS marker genes (86). In both of these cases, the sequencing depth is effectively reduced, diminishing the power to detect genuine differences in the microbiome.

Most strains used in probiotics do not appear to become permanent colonizers of the gut (86–88). Therefore, we recommend withholding probiotics for a minimum of 48 h prior to fecal sampling for microbiome testing to allow a full transit time for all material in the gut. If probiotics are an integrated component of the intervention or formulation being tested, we recommend creating an alternate formulation that is otherwise identical, but without probiotics, for use during this period. We note that fungal probiotics will not interfere with bacterial marker gene sequencing, nor bacterial probiotics with fungal marker gene sequencing.

### Comparing to a “Normal” Microbiome

When conducting research to support statements about the gut microbiota and health, it is critically important to consider what the reference is for a “normal” or a “healthy” microbiome (89). Consumers often wish to know if their pet is healthy or normal, and are concerned with feeding a health-promoting diet to their pet (90). For researchers and commercial companies interested in the microbiota, being able to make a product claim like “supports a healthy gut microbiota” may be a desired goal in order to attract these customers. However, defining a normal, healthy microbiome is a non-trivial task that has been a topic of much debate [reviewed in (91, 92)], and encompasses both the taxonomy and the function of microbial communities.

Within the human gut microbiome, it is generally agreed that the community is more stable at the level of functions encoded in genomes than at the taxonomic level, but at higher taxonomic levels the structure and composition of microbiomes are largely preserved across individuals (8, 93, 94). Many microbiome studies remain limited to marker gene surveys that cannot ascertain functional information, thus we limit our discussion here to the taxonomic definition of the normal microbiome.

Using a small number of individuals, or animals from a single research colony or location is not sufficient, as demonstrated by the large effects of research facility in mice (95) and primates (96), and the large variation across studies in companion animals (97). Ideally, sampling of many individuals of diverse genetic backgrounds from multiple locations is suggested (89). If carefully selected, publicly available sequence data from other studies can be included (where similarity of methods allows) to expand their definition of healthy or normal in a study; see (98) for an example of how data from multiple studies can be combined.

Reference data sets for the human microbiome range in scale from several hundred (94, 99) to over 10,000 individuals (100). The smaller of these reference sets did not fully sample the diversity of the microbial population [for example, compare (94) to (99)]. The gut microbiota of dogs are relatively similar to those of humans (101), suggesting that samples from hundreds or thousands of individuals may be necessary to understand the healthy microbiota in companion animals, and that commonly utilized sample sizes in companion animal research are not sufficient for this purpose.

While a publicly available reference data set currently doesn't exist for normal or healthy dogs and cats, there is an accumulation of evidence that some taxa are commonly observed in high relative abundance in the gut microbiota of companion animals. When many of these taxa are missing or present in only trace numbers, it is more likely that the important functions normally performed by the gut microbiome are impaired, and that the microbiota are in a state of dysbiosis (102). Several disease states are associated with high abundances of specific taxa, the absence of key taxa, or dysbiosis generally [e.g., (14, 15, 102–104)]. In summary, it is easier to define an unhealthy microbiome than a healthy one (92) and we have identified a need for a reference data set of microbiomes from a large and diverse group of healthy companion animals.

### Data Analysis

While the best practices for analyzing study results are out of scope for this review, we highlight several considerations for this stage of a study. Statistical tests are important to determine if results are conclusive and to back product claims. Which statistical test(s) to use depend on the data that is being analyzed. For example, a statistical analysis of a study with a parallel design (105) will be different from a statistical analysis of a study with a crossover design (106), which requires samples from the same animal to be grouped differently (called a random factor). We refer readers to several resources to determine the appropriate statistical test(s) and analysis for a study (37, 107–109).

Different methodological approaches yield data sets of varying complexity, size and information content. We advise researchers to have a firm understanding of the type of data generated, how to analyze it, and whether it will fully address research questions and hypotheses. The analysis of some data types, such as metagenomics or shotgun sequencing, are highly complex and require an experienced bioinformation to analyze the results (33). Time, budget, and expertise for data analysis should be carefully considered and is sometimes overlooked in study design.

## Dietary Considerations

There are several aspects of diet formulation and diet changes that should be considered in microbiome studies that differ from traditional feeding trials. Our recommendations in this area are generally focused on reducing variability between individual animals and over time in the same animal in order to facilitate the detection of genuine differences. For studies that involve non-dietary interventions, we suggest that researchers be aware of how dietary choices can influence the microbiota, and consider controlling for diet or at minimum, recording and reporting dietary information. While this section is focused on nutritional studies, several of the recommendations are still valid considerations for clinical trials.

### Uniform Pre-study Diet

As established by many other studies, diet significantly affects the microbiota in companion animals (58, 110). A dietary change will often lead to microbiota changes, but this response is individualized and depends in part on the pre-existing microbiota (111). Therefore, it is important to note that the microbiota (including overall diversity and relative abundance of specific taxa) of different individuals can change in different ways when exposed to the same diet (112–115). If the animals used in a study have been eating a variety of diets prior to the start of the study, this can introduce additional variability to the feeding trial. The effect of this can be reduced by feeding a uniform pre-study diet to animals in the study, which we recommend doing for a minimum of 30 days. It is often pragmatic to use the control diet for the study as the pre-study diet, particularly if a small effect size is predicted for your treatment. This can help to reduce variability in the control group or period.

### Pre-testing of the Diet

For certain types of diets we recommend microbiome testing of the food itself prior to the study and/or careful monitoring of the manufacturing of the food used in your study. While manufacturers must label pet food with the guaranteed analysis for minimum percentages of crude protein and crude fat, and maximum percentages of crude fiber and moisture, this requirement does not include prebiotics and probiotics. Further, Olivry and Mueller (116) found that many pet foods contain undisclosed ingredients. Manufacturers of commercially available pet supplements are not required to disclose the presence of GRAS (generally recognized as safe) ingredients that comprise <1% of the product on the product label, including probiotics (117). Under these regulations, it is possible for commercial foods to contain prebiotics and probiotics that are not listed in the ingredients, but that may interfere with microbiome testing. Pet foods that do list probiotic ingredients do not always contain the stated organisms, and may contain other organisms that are not listed (118). Other types of foods including fresh and raw foods have a higher risk of microbial contamination and transmission of potential pathogens than canned or extruded preparations (119–121). For these reasons, the best practice is to conduct microbiome testing of all commercially available foods, as well as fresh and raw foods, before beginning a study to account for any microbial signals that may arise from the foods themselves.

### Limit Dietary Variables

When designing or selecting diets to be fed during the trial, the key variable of interest should be identified and only this variable should be manipulated if possible. For example, keeping macronutrient ratios, ingredients, and preparation methods the same between diets wherever possible helps to ensure that observed effects are due to the intervention of interest. To illustrate this point, Hall et al. (12) utilized diets with differing fiber sources, that were otherwise nearly identical in macronutrient concentration and very similar to the pre-trial food. Carmody et al. (122) tested the effect of cooking foods on the microbiota using identical foods differing only in the preparation method.

### Testing on Multiple Levels

If there is a sufficient number of animals in the study, multiple levels of the factor of interest can be tested to provide more robust evidence of an effect. For example, four different percentages of cricket protein from 0 to 24% were tested in Jarett et al. (65), which observed a gradient in the relative abundance of several sequence variants with increasing concentrations of cricket protein. This can be a useful approach for several reasons. First, observing microbial taxa that increase or decrease in correlation to an ingredient provides a more convincing result. This approach could also be used to help determine the optimal amount of a dietary component from a microbial perspective, especially for a costly ingredient.

## Recruitment

Recruitment of companion animals is one of the most crucial phases of a study, and one where poor choices or unanticipated confounding factors can result in unusable results. Fortunately, many of these factors can be anticipated and used as inclusion or exclusion criteria. As much information as is available should be recorded about the recruited animals including: the geographic location of the animal, whether it shares a household with other animals in the study, its diet prior to the study (including treats and other supplementary foods), breed, age, neuter status, veterinary diagnoses, medications, supplements, and any antibiotic use within the last 6–12 months. Research on the human microbiome has confirmed the importance of recording this and analogous information in human studies (89). While some factors can be assessed without testing or examining the individual animal, there are other steps that should be taken to ensure all inclusion criteria are met.

Which animals to enroll in a study varies greatly with the goal of the study. In all cases, we recommend excluding animals that are outliers whether it be from a health condition perspective or microbiota composition perspective. In particular, we recommend that all animals in a study be in a similar life stage, with animals in young to middle adulthood being preferable to juvenile, senior, or geriatric animals. This is because the microbiota of companion animals undergoes profound and often rapid shifts during development from birth to adulthood (4, 78, 79), and microbial diversity and composition are significantly altered in senior and geriatric dogs (123, 124) as well as in humans and mice (125, 126). In older individuals, the metabolic activity of the gut microbiota may be reduced (127), and shifts in specific taxa may lead to a pro-inflammatory state (125). Recruiting animals at a similar life stage increases the likelihood that their microbiota and immune status are functionally similar.

### Pre-screening

While additional pre-screening measures can be costly, they are critical to ensure that all inclusion criteria are met for animals recruited for a study. Without adequate pre-screening, particularly for microbiome studies, the success of the entire study can be jeopardized by inconclusive results. Furthermore, pre-screening can serve as a baseline of comparison for a post-study veterinary exam and microbiome test to measure any health improvements. There are situations where pre-screening the microbiome of individuals in the study is not necessary. For instance, if a diet or intervention is targeted toward animals with a specific health issue that is associated with the microbiota such as chronic enteropathy, most animals in the study will likely be dysbiotic (16, 102, 128).

Veterinary exams are a useful method for pre-screening animals and ensuring that health related criteria are met. While colony animals typically receive frequent veterinary exams and health problems are detected early, health problems in pets are common and not always known to the owner. In one analysis of over 7,000 dogs, approximately one in three pet dogs have signs or risk factors for health problems (129). Aside from possibly increasing variation in the response to diet, these occult health problems might increase the chance of adverse reactions to the diet or intervention, thus increasing the dropout rate. As a benefit, veterinary exams offer the opportunity to assess health metrics such as serum chemistry, skin condition, weight, and more alongside the microbiota response when they are conducted both before and after a trial.

We strongly recommend pre-screening the gut microbiome of all individual animals before they are enrolled in a study. First, this can identify dysbiotic animals or those with highly imbalanced microbiomes who otherwise meet all inclusion criteria. Multiple gastrointestinal disorders, such as chronic enteropathy (CE) and inflammatory bowel disease, are associated with dysbiosis in companion animals (102). These disorders are common in both cats and dogs, with a reported prevalence up to 17.8% (130, 131); a microbiome test can identify asymptomatic animals with dysbiosis, as microbial imbalances do not necessarily resolve when symptoms do (15, 132). We recommend excluding animals with dysbiosis from feeding studies in particular, because diet changes can trigger the exacerbation of previously asymptomatic or subclinical health problems for which the dysbiotic microbiome was an early indicator. For example, animals with dysbiosis may be more prone to food-responsive diarrhea (14, 133). These dysbiotic animals may still be suitable for studies on non-dietary interventions, depending on the intervention and the study goals.

There are several specific types of microbial imbalances that we recommend as exclusion criteria. These include: high relative abundances of frank or opportunistic pathogens such as *Escherichia coli* (134), *Campylobacter* (135, 136), or *Clostridioides difficile* (137, 138); elevated levels of bacteria typically found in the small intestine such as *Streptococcus* or *Veillonella* (139, 140); elevated levels of bacteria associated with inflammatory bowel disease such as *[Ruminococcus] gnavus* group (141) or *Desulfovibrio* (142); relative abundances >10% of genera that are not usually found in companion animals in high abundance; and relative abundance of a single genus >50%. While these are our current best recommendations, they can change with scientific advancement. In this perspective, researchers should have an understanding of normal and abnormal microbiomes of animals in their population base and use their best judgement for recruitment decisions.

### Using Pets vs. Colony Animals

One of the pivotal decisions that researchers are faced with when recruiting companion animals for a study is whether to use animals from a research colony or household pets. In this section, we outline the benefits and drawbacks of both options to help researchers make informed decisions about how to recruit for a study. A summary of the side-by-side comparison of using colony animals to pets is outlined in Table 1.

**Table 1:** Comparison of using colony animals or household pets in an intervention study.

	**Colony animals**	**Pets in homes**
**Key attributes**	Genetically homogenous, particularly for dogs Similar living environment Limited number of pre-study diets (usually) Known health status of animals, usually healthy Possibility of exchange of microbes between individuals	Genetically diverse Wide variety of environments Many different pre-study diets Variety of states of health, known and unknown Different body condition scores, many overweight or obese
**Pros**	Easy and fast to recruit Excellent compliance with study protocol, sample collection, monitoring Recent veterinary exam (usually) Very low dropout rate Less inter-individual microbiome variability	Lower cost Results are more generalizable to pet population Results may be more replicable in other pets Higher likelihood of ethical acceptability to consumers Can be a tool to increase customer engagement
**Cons**	Higher cost Results may be less generalizable to pet population Results may be less replicable due to facility and vendor effects	Slower and more expensive to recruit Lower and more variable rates of compliance with study protocol Prolonged, complex protocols or those requiring a veterinarian may not be possible Higher dropout rate (especially cats) More inter-individual microbiome variability

*Time, budget, access, research question(s), type of intervention and so on are all factors to consider when recruiting companion animals for a study*.

#### Colony Animals: Benefits

We will first consider the advantages of using animals from research colonies for intervention trials that study the microbiome. A facility will have many animals, making recruitment easy and rapid. Animals will often have a recent veterinary exam already recorded, and the dropout rate is typically very low. Facility staff will ensure that there is a high rate of compliance with study protocols including diet changes, sample collection, sample storage, and reliable & accurate monitoring of signs (e.g., fecal score, amount of food consumed).

In regard to microbiome studies, there is typically less variability between the microbiomes of individual animals from the same colony than would be observed in the same number of pets, for several reasons. First, colony animals tend to be of the same breed, within a similar age range, of a healthy body condition score, and in good health, all of which contribute to more consistent microbiomes (8, 123, 143, 144). Second, there are multiple environmental factors that act to homogenize their microbiomes, including similar housing conditions, consuming the same diet prior to the study, indirect microbial exchange through shared handlers, and in some cases direct microbial exchange in shared spaces or co-housing (145). Together, all of these factors can result in reduced variability and increased power to detect differences due to study treatments. This makes colony animals an excellent choice when the effect size of the intervention is anticipated to be small.

#### Colony Animals: Drawbacks

There are several drawbacks in using colony animals, however, which are primarily related to cost, and the generalizability & replicability of study results (146). Colony animals are typically more expensive than pets, even when recruitment services for pets are included in costs. The same aspects that make colony animals less variable also make results of these studies less generalizable to the overall population of pets. Pets comprise many different breeds, all life stages, and many have health problems including overweight and obesity (90, 129). This diversity is an important consideration because many diets and products are designed for and marketed to pets.

Microbiome studies on other laboratory animals, including rodents and primates, suggests that results obtained from microbiome studies on colony animals might be difficult to replicate with animals from a different colony. Animals from different vendors or facilities, even if they are extremely similar genetically, have different microbiomes (95, 96, 147). The “vendor effect” can be larger than the effect of diet (95) and can impact multiple body systems (e.g., the immune system) (148, 149). In response to these findings, multiple papers have been published recommending mitigation measures and metadata to collect (143, 150), some of which are applicable to companion animals. Although this has not been specifically studied in companion animals, we believe the principles and driving forces are similar to those in rodents, and that researchers should be alert to a similar phenomenon in companion animals from research facilities.

#### Household Pets: Benefits

While using household pets can present some unique challenges to microbiome studies, they can offer a more generalizable and cost-effective result that is more likely to be confirmed by customers' experiences. Pets are genetically diverse, vary in age and health status, live in a variety of different environments, and consume many different diets so the results are correspondingly more likely to translate to the full pet population. The cost of the study is likely to be less than a study with colony animals, depending on the services and incentives that are provided to pet owners as part of the study.

Customers are both pet owners and consumers, which should be considered in recruitment decisions. As pet owners, they may find animal research that takes place in centralized facilities using dedicated animals to be morally objectionable. Therefore, in-home studies on pets are more likely to be viewed as ethically acceptable (151) and these results can be publicized to customers more easily. As consumers, they will have increased engagement with a brand or product through study participation, which has been demonstrated by the popularity of various “citizen science” initiatives (100, 152).

#### Household Pets: Drawbacks

There are limitations to using pets at all stages of research, from recruitment, to study design, to interpretation of results. Recruitment of pets is slower and more involved than for colony animals, and additional considerations are required for a successful study. We have found social media to be a helpful tool for recruitment and retention, especially when pet owners are informed about the study aims, educated about the importance of following the study protocol, and emotionally invested in the study results. Incentives, financial or otherwise, are also important for recruitment and retention purposes. Despite the ease of recruiting multiple pets per household, we do not recommend this practice. Pets in the same household experience very similar conditions, are exposed to the same environmental microbes, and usually have ample opportunity to exchange microbes with each other, so they do not represent independent samples. This is analogous to the cage clustering effect in mice (153), where samples must be considered as clustered for analysis, reducing statistical power (154).

Some design choices are not compatible with the use of pets. For instance, if a diet is intended to help a health condition, pet owners are unlikely to be willing to forego other treatments that might help their pet for the period of the study. Similarly, owners may not want to participate in a prolonged study (in our experience, longer than ~60–90 days), and may not be able to reliably execute a complex experimental or sampling protocol. Sampling protocols that require a veterinary professional (e.g., for serum collection), onerous sample storage, and shipping requirements (e.g., frozen samples) are more difficult to carry out with pets.

When pets are used, it is very helpful to have a person or team available to respond to owners' questions and concerns as they arise, and to proactively check in as the study progresses. A substantially higher dropout rate should always be anticipated in pets. This problem is particularly acute for feeding studies that include cats, largely due to palatability issues (155), and for any diet or treatment that targets a health concern. While many pet owners involved in studies make their best efforts to comply with study protocols, compliance is likely to be imperfect, which will contribute to variability in microbiota response. The inherently higher variability in pet microbiota and the different pre-study diets of pets increase the importance of a consistent pre-study washout diet wherever feasible. It may be harder to detect differences in the results of the study due to the variability within the study cohort, but this can be ameliorated with a large sample size.

## Key Take-Aways

“Cheap is expensive, and expensive is cheap”: an adage that couldn't be more applicable to investing in pet microbiome research. Limiting study costs poses a substantial risk of conducting an underpowered or fundamentally flawed study with inconclusive results. Conversely, a larger expenditure for a well-designed study of adequate size may be more expensive up front, but can pay dividends in credible, well-supported product claims and more successful products. This manuscript outlines the best practices for conducting successful studies, even for those with smaller budgets. Our strongest recommendations for best practices in companion animal studies with microbiome testing are to simplify the research question(s), perform microbiome pre-screening, include appropriate controls, consider a consistent pre-study diet for all animals, and allow 30 days for full microbiome response to diet change.

We emphasize the importance of microbiome testing in intervention studies not only to generate and/or validate product claims, but also as a screening tool for recruitment and benchmarking purposes. Microbiome testing is a useful tool in veterinary medicine as well. There are many diseases associated with gut imbalances, some of which can be detected with microbiome testing even before a patient becomes symptomatic. Furthermore, testing before treatment provides an important benchmark to determine if interventions are effective. We advise veterinarians to stay current on microbiome studies, as evidence-based practices and microbiome testing in intervention trials are becoming more common to support product claims.

## Author Contributions

All authors contributed to the writing, editing, and revising of the article and approved the submitted version.

## Conflict of Interest

The authors of this article work for AnimalBiome, a company that sells microbiome testing services; microbiome testing is mentioned as one of many recommendations the authors outline in this review.
